# A revised dated phylogeny of the arachnid order Opiliones

**DOI:** 10.3389/fgene.2014.00255

**Published:** 2014-07-28

**Authors:** Prashant P. Sharma, Gonzalo Giribet

**Affiliations:** ^1^Division of Invertebrate Zoology, American Museum of Natural HistoryNew York, NY, USA; ^2^Museum of Comparative Zoology, Department of Organismic and Evolutionary Biology, Harvard UniversityCambridge, MA, USA

**Keywords:** phylogenomics, arachnids, arthropods, molecular dating, total evidence dating, node dating

## Abstract

Dating the Opiliones tree of life has become an important enterprise for this group of arthropods, due to their ancient origins and important biogeographic implications. To incorporate both methodological innovations in molecular dating as well as new systematic discoveries of harvestman diversity, we conducted total evidence dating on a data set uniting morphological and/or molecular sequence data for 47 Opiliones species, including all four well-known Palaeozoic fossils, to test the placement of both fossils and newly discovered lineages in a single analysis. Furthermore, we investigated node dating with a phylogenomic data set of 24,202 amino acid sites for 14 species of Opiliones, sampling all extant suborders. In this way, we approached molecular dating of basal harvestman phylogeny using different data sets and approaches to assess congruence of divergence time estimates. In spite of the markedly different composition of data sets, our results show congruence across all analyses for age estimates of basal nodes that are well constrained with respect to fossil calibrations (e.g., Opiliones, Palpatores). By contrast, derived nodes that lack fossil calibrations (e.g., the suborders Cyphophthalmi, and Laniatores) have large uncertainty intervals in diversification times, particularly in the total evidence dating analysis, reflecting the dearth of calibration points and undersampling of derived lineages. Total evidence dating consistently produced older median ages than node dating for ingroup nodes, due to the nested placement of multiple Palaeozoic fossils. Our analyses support basal diversification of Opiliones in the Ordovician-Devonian period, corroborating the inferred ancient origins of this arthropod order, and underscore the importance of diversity discovery—both paleontological and neontological—in evolutionary inference.

## INTRODUCTION

Dating molecular phylogenies has the power to provide an evolutionary framework for a group in question, beyond inference of relationships alone (Benton, 1995; Wang et al., 1999). Therefore, dating phylogenies has become a standard practice in phylogenetic, biogeographic, ecological, and other evolutionary studies (Pisani et al., 2004; Benton and Donoghue, 2007; Donoghue and Benton, 2007; Laurin, 2012). Several methods exist to calibrate the molecular “clock” (Zuckerkandl and Pauling, 1962) such as biogeographic calibration points and fossils, the advantages and drawbacks of each methodology having been discussed elsewhere (e.g., Kodandaramaiah, 2011). The fossil record has also been used in a diversity of manners, either to constraint nodes (“node dating”), or using fossils as terminals in combined analyses of morphology and molecules (“total evidence dating”; Murienne et al., 2010; Pyron, 2011; O’Leary et al., 2013). The effects of both practices are just beginning to be evaluated (Wood et al., 2013), but is clear that special attention should be paid to the actual fossils used for calibration, as precise dating and accurate phylogenetic placement of the fossil material has important effects on the final results of the analyses, demanding standards that had been largely ignored until recently (Parham et al., 2012). Another key issue is incorporation of uncertainty into calibrations (Ho and Phillips, 2009), as recently demonstrated in an analysis of the biogeographically iconic trees of the genus *Nothofagus* (Sauquet et al., 2012). Consequently, recent studies have evaluated a range of dating techniques and calibrations to better test biogeographic hypotheses (Mao et al., 2012; Murienne et al., 2014), a practice that should become more widespread in future investigations of evolutionary history.

A separate concern for molecular dating is confidence in the adequacy of taxonomic sampling, which affects topological accuracy as well as precision of molecular divergence times. Particularly for hyper-diverse clades, such as arthropods or flowering plants, continually accruing knowledge of biodiversity results in periodic amendments to systematics and phylogeny. Specifically, the unanticipated discovery of a proximal basally branching group with respect to a clade of interest will increase that clade’s diversification age, assuming that the discovery does not change interpretation of fossil placements. Furthermore, simultaneous discovery of new fossils in tandem with systematic rearrangements of extant species necessitates wholesale reanalysis of a given taxon’s diversification history.

**FIGURE 1:**
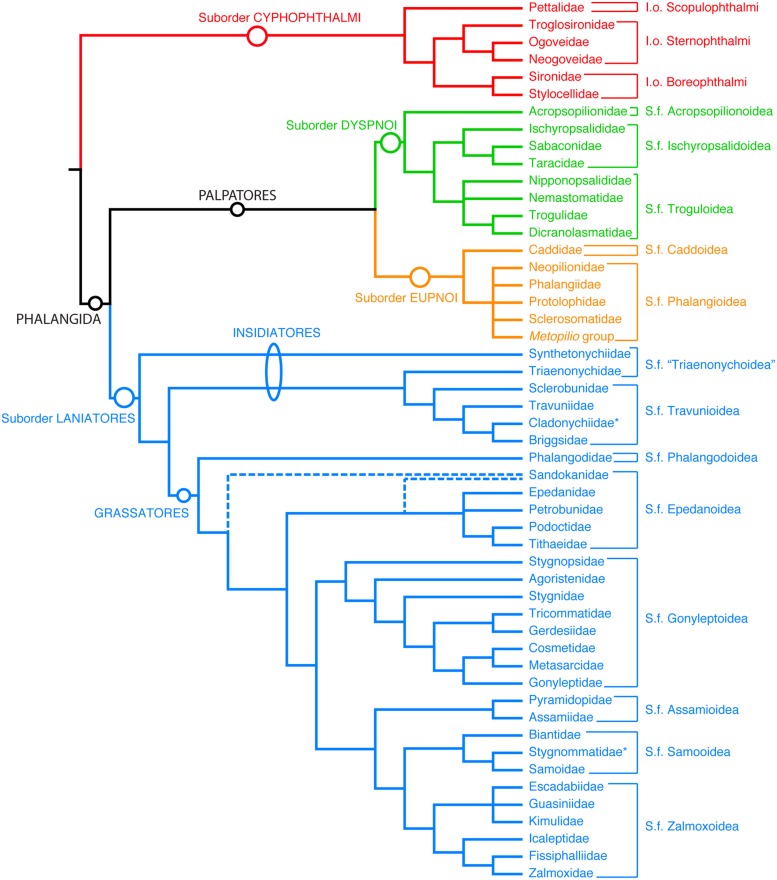
**Summary tree of the phylogenetic relationships of the extant Opiliones families.** I.o, infraorder, S.f, superfamily. Unranked clades are indicated to facilitate discourse.

One such taxon is the arachnid order Opiliones (harvestmen or daddy-long-legs, among other common names; **Figure 1**), one of the best-studied arthropod groups in terms of molecular phylogenetics, and more recently, the timing of cladogenesis (Giribet et al., 2010, 2012a; Sharma and Giribet, 2011; Hedin et al., 2012; Garwood et al., 2014). In contrast to many terrestrial arthropod orders, Opiliones is a group with a Palaeozoic fossil record, and for which morphological matrices coding these fossils are available (Giribet and Dunlop, 2005; Garwood et al., 2011, 2014). Modern-looking members of the suborders Eupnoi and Dyspnoi were already present in the Carboniferous (Garwood et al., 2011). Furthermore, a specimen previously placed in Eupnoi is known from the Devonian Rhynie Cherts (Dunlop et al., 2003, 2004). This fossil, *Eophalangium sheari*, has been used in recent studies to constraint the age of Eupnoi or Palpatores in previous dated phylogenies (Giribet et al., 2010; Hedin et al., 2012), based upon interpretation of individual characters; none of these studies included *E. sheari* as a terminal in a phylogeny. The recent discovery of a new Opiliones fossil in the Carboniferous Montceau-les-Mines (Garwood et al., 2014), and its sister group relationship to the Devonian *E. sheari* necessitated re-evaluation of the Opiliones dated phylogeny (Garwood et al., 2014), specifically due to the unexpected placement of this fossil clade sister to Cyphophthalmi, and not Eupnoi or Palpatores. Node dating of a 279-taxon dataset in that study suggested a very different scenario for the timing of subordinal diversification (Garwood et al., 2014).

Concomitantly, phylogenetic reassessment of extant Opiliones has greatly changed the understanding of the group’s basal relationships. In addition to the discovery of the extinct sister group of Cyphophthalmi (Garwood et al., 2014), recent molecular phylogenetic efforts have revealed an unknown sister group of the suborder Dyspnoi, the resurrected family Acropsopilionidae (Groh and Giribet, 2014), indicating an older basal divergence of this lineage than estimated heretofore. This parallels the identification of Synthetonychiidae, a relictual family endemic to New Zealand, as the sister group of the remaining Laniatores (Giribet et al., 2010; Sharma and Giribet, 2011), and definitive placement of Pettalidae, a temperate Gondwanan family, as the sister group of the remaining Cyphophthalmi (Giribet et al., 2012a). Thus, discoveries of fossils and new, refined phylogenetic hypotheses in the last five years necessitate further reevaluation of Opiliones diversification and divergence time estimation.

To incorporate (a) methodological innovations in molecular dating, (b) discoveries of new fossils and fossil placements, and (c) systematic rearrangements of extant harvestman diversity, we conducted the first total evidence dating analysis for harvestmen, uniting morphological and/or molecular sequence data for 47 Opiliones species, including all four well-known Palaeozoic fossils and two Mesozoic fossils. We thus tested both the placement of fossils and the newly discovered extant lineages in a single analysis. We separately conducted node dating on this data set after culling fossil taxa, to examine the effect of treating fossils as terminals. To approach node dating with a modern data set, we amassed a phylogenomic data set of 24,202 amino acid sites for 14 species of Opiliones, sampling all extant suborders. In this way, we approached comparative molecular dating of basal harvestman phylogeny using different data sources and approaches to assess congruence of divergence time estimates.

## MATERIALS AND METHODS

### TOTAL EVIDENCE DATING

An existing morphological matrix of 158 unordered characters recently analyzed by the authors (Garwood et al., 2014) was modified for total evidence dating. To this matrix, we added exemplars of Pettalidae (*Pettalus thwaitesi*), Caddidae (*Caddo pepperella*), Acrosopilionidae (*Acropsopilio neozealandiae*, *Austropsopilio altus*, and *Caddella croeseri*), and a new lineage of Phalangioidea not ascribed to a family (*Hesperopilio magnificus*), thus improving sampling of basally branching lineages in Cyphophthalmi, Eupnoi, and Dyspnoi, respectively. Synthetonychiidae, the sister lineage of the remaining Laniatores, could not be included in this matrix, as a previously sequenced species was small and was entirely consumed in previous DNA extractions (Sharma and Giribet, 2011). The modified matrix was 85.7% complete, and 10.5% of the total matrix consisted of inapplicables. This morphological matrix was combined with molecular sequence data from five genes, totaling 3,885 aligned nucleotide positions (sequence data previously published; Giribet et al., 2002, 2010; Sharma and Giribet, 2011; Groh and Giribet, 2014). The combined alignment is provided as supplementary material.

We also changed outgroup sampling in this matrix, replacing the troglomorphic scorpion *Belisarius xambeui* with the more representative *Pandinus imperator* (both exemplars of Iurida). This was done to reduce ambiguity in optimization of characters pertaining to eyes, as almost all scorpions bear multiple pairs of eyes, with a few exceptional troglomorphic species like *B. xambeui* (Prendini et al., 2010). This is pertinent to the placement of the harvestman fossil *Hastocularis argus*, the only Opiliones interpreted to bear two sets of eyes (Garwood et al., 2014). The final matrix included four outgroup terminals, 41 extant harvestman terminals (five Cyphophthalmi, eleven Laniatores, eleven Eupnoi, and fourteen Dyspnoi), and six extinct harvestman terminals (four Palaeozoic, two Mesozoic). Priors of calibration points used in the analyses are provided in **Table 1**.

**Table 1:** Priors utilized for node calibrations in molecular dating analyses.

Node	Total evidence dating	Combined matrix node dating	PhyloBayes, LN model	PhyloBayes, UGAM model
Arachnida	Uniform (465–495)	Uniform (465–495)	Uniform (465–495)	Uniform (465–495)
Opiliones		Exponential (mean 425, offset 411)	Soft upper bound only (minimum 411)	Soft upper bound only (minimum 411)
Eupnoi		Exponential (mean 320, offset 305)	Soft upper bound only (minimum 305)	Soft upper bound only (minimum 305)
Dyspnoi		Exponential (mean 320, offset 305)	Soft upper bound only (minimum 305)	Soft upper bound only (minimum 305)
Araneae			Soft upper bound only (minimum 305)	Soft upper bound only (minimum 305)

Total evidence dating made use of the independent gamma rates model. *Limulus polyphemus* (Xiphosura) was used to root the tree. For molecular sequence data, we excluded third codon positions of the genes cytochrome *c* oxidase subunit I and histone H3 in order to retain conserved sites only. A one-parameter Markov model (Lewis, 2001) was applied to the morphological data. A unique GTR model of sequence evolution, correction for a discrete gamma distribution, and a proportion of invariant sites (GTR + Γ + I) were specified for each molecular partition, as selected in jModeltest v. 0.1.1 (Guindon and Gascuel, 2003; Posada, 2008) under the Akaike information criterion (Posada and Buckley, 2004). Analyses followed the methods deployed for a hymenopteran data set by Ronquist et al. (2012a) using MrBayes v.3.2.2 (Ronquist et al., 2012b). In summary, we compared branch lengths from non-clock and uncalibrated strict clock analyses, and used the slope of the variance as the median for an exponential hyperprior for variance increase. A prior for the substitution rate was obtained by dividing the median tree height from uncalibrated strict clock analyses with an estimated age of the Xiphosura-Arachnida split. We used 475 Myr as an approximation of the latter, as inferred from the early fossil record of Euchelicerata (reviewed by Dunlop, 2010). Dividing the tree height by the age of this split resulted in an estimated clock rate of ∼5.103 × 10^-4^ substitutions per site per million years, which was used as the mean of a lognormal prior on the clock rate. The standard deviation of the lognormal was also chosen following the method of Ronquist et al. (2012a), such that dividing the upper 95% estimate of the tree height by 475 Myr was displaced by one standard deviation from the mean. Uncertainty in the ages of fossils was disregarded, as it was considered negligible with respect to other sources of error and the scale of geological time spanned by Opiliones. To bound the root age of the tree and maintain consistency with the fossil record, a uniform prior of 465–495 Ma was applied to the divergence of Arachnida (Dunlop, 2010; Garwood et al., 2014).

Four runs, each with three hot and one cold Markov chains, were executed for 2 × 10^7^ generations. Convergence diagnostics were assessed using Tracer ver. 1.5 (Rambaut and Drummond, 2009) and 25% of each run was discarded as burnin.

### NODE DATING WITH COMBINED MORPHOLOGICAL AND MOLECULAR DATA SET

To compare the effects of total evidence dating versus node dating on the combined data set, the combined data matrix from the total evidence analysis was analyzed with fossils removed. Partitioning and models applied to the morphological and molecular data sets, as well as independent gamma rates model priors, were as specified in the total evidence analysis. A uniform prior of 465–495 Ma was applied to the divergence of Arachnida (Dunlop, 2010; Garwood et al., 2014), as in the total evidence dating. Based upon the placements of Palaeozoic harvestman fossils in the total evidence topology, three node calibrations were applied as exponential prior distributions, thus permitting node ages only to exceed a given minimum age constraint. The age of Opiliones was constrained with an offset (lower bound) of 411 Ma and a mean of 425 Ma, reflecting the age of *E. sheari*. Both Eupnoi and Dyspnoi calibration priors consisted of exponential distributions with an offset of 305 Ma and mean of 320 Ma, reflecting the ages of *Ameticos scolos* and *Macrogyion cronus*.

Four runs, each with three hot and one cold Markov chains, were executed for 2 × 10^7^ generations. Convergence diagnostics were assessed using Tracer ver. 1.5 (Rambaut and Drummond, 2009) and 25% of each run was discarded as burnin.

### NODE DATING WITH PHYLOGENOMIC DATA SETS

Transcriptomic data for several species of Opiliones were analyzed previously as part of a broader investigation of arachnid relationships (Sharma et al., in review). From a supermatrix of 3,644 orthologs and >1.2 million aligned amino acid positions, we retained the following outgroup taxa for analysis: one Xiphosura (*Limulus polyphemus*), two Scorpiones (*Centruroides vittatus*, *Pandinus imperator*), five spiders (*Liphistius malayanus*, *Acanthoscurria gomesiana*, *Frontinella communis*, *Leucauge venusta*, *Neoscona arabesca*), one Amblypygi (*Damon variegatus*), and one Uropygi (*Mastigoproctus giganteus*). Retained ingroup taxa consisted of all 14 Opiliones transcriptomes sequenced to date (two Cyphophthalmi, six Laniatores, three Eupnoi, three Dyspnoi). Subsequent to reducing indel-containing sites with GBlocks v.0.91b (allowing gap positions if occupying half of a column or less; Castresana, 2000), the resulting alignment consisted of 24,206 positions and 14.07% missing data.

Divergence times based on this alignment were inferred using PhyloBayes v.3.3f (Lartillot et al., 2009). A constraint tree was provided, based on analyses of the original data set (Sharma et al., in review). *Limulus polyphemus* was used to root the tree as in other analyses. The split between Xiphosura and Arachnida was again constrained with a uniform prior of 465–495 Ma. As PhyloBayes can implement soft bounds, we constrained the floor of Opiliones as 411 Ma (based on the age of the oldest fossil, *E. sheari*), the floor of the represented Dyspnoi divergence as 305 Ma (based on the age and placement of the fossil *A. scolos* as crown-group Dyspnoi in the total evidence topology; Acropsopilionoidea not included in the analyses), the floor of the represented Eupnoi as 305 Ma (based on the age of the fossil *Macrogyion cronus*; Caddoidea not represented either), and the floor of basal spider divergence as 305 Ma (based on a mesothele spider fossil of Carboniferous age, from the same deposit as the Carboniferous harvestmen; Dunlop, 2010). The ceilings of these four nodes were not bounded.

Lognormal (LN) and uncorrelated gamma multipliers (UGAM) clock models were implemented with these constraints. Four runs were conducted under each model for 14,967–19,357 cycles. For all runs, 5000 cycles were discarded as burnin.

## RESULTS

### TOTAL EVIDENCE TOPOLOGY AND DATING

**FIGURE 2:**
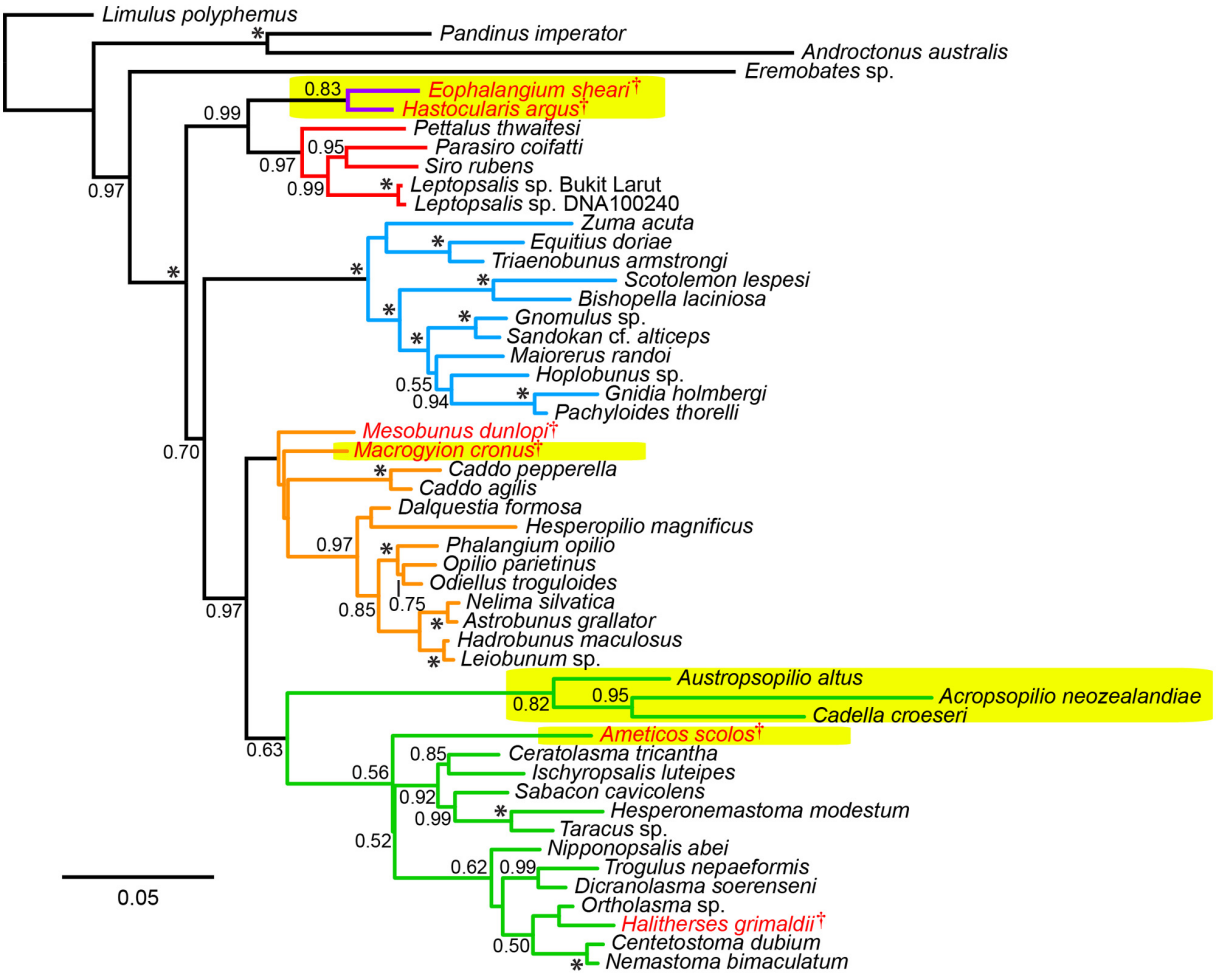
**Uncalibrated total evidence tree topology.** Colors in tree correspond to suborders (purple: Tetrophthalmi; red: Cyphopthalmi; blue: Laniatores; orange: Eupnoi; green: Dyspnoi). Fossil taxa indicated in red lettering. Major recent amendments to Opiliones systematics are indicated in yellow highlights: discovery of Tetrophthalmi, the stem group of mite harvestmen; discovery of *Macrygyion cronus* and *Ameticos scolos*, the oldest crown group Eupnoi and Dyspnoi, respectively; and placement of Acrosopilionidae as basally branching Dyspnoi. Asterisks indicate posterior probabilities of 1.00.

Bayesian inference analysis of the total evidence data set recovered a consensus tree topology supporting previous understanding of Opiliones relationships (**Figure 2**). Specifically, monophyly of Opiliones and its four suborders, as well as the higher clades Phalangida (Dyspnoi + Eupnoi + Laniatores) and Palpatores (Eupnoi + Dyspnoi; **Figure 1**), was obtained with a posterior probability (PP) of 1.00. In accordance with the systematic treatment of Groh and Giribet (2014), *Hesperopilio* was recovered as nested within Phalangioidea (PP = 1.00); Caddidae *sensu stricto* was composed of only the genus *Caddo*, the sister group to the remaining Eupnoi; and Acropsopilionidae was recovered as sister group to the remaining Dyspnoi.

Fossil placements within the tree topology were consistent with the total evidence analysis of Garwood et al. (2014), albeit with significant nodal support for the placement of Tetrophthalmi as sister group to Cyphophthalmi (PP = 0.99), due to the substitution of the blind scorpion outgroup exemplar *Belisarius xambeui* with the more typical representative of Iurida, *Pandinus imperator*, in the total evidence matrix. This result indicates that the analysis of Garwood et al. (2014) underestimated support for the Tetrophthalmi + Cyphophthalmi relationship, due to choice of an atypical outgroup species. By contrast, the placements of *Ameticos scolos* and *Macrogyion cronus* within Dyspnoi and Eupnoi, respectively, were not supported (as in the previous analysis of Garwood et al., 2011). *Ameticos scolos* resolved as sister taxon to either Acropsopilionidae or Ischyropsalidoidea + Troguloidea in the post burnin trees. *Macrogyion cronus* is very likely a member of Phalangioidea, but is difficult to place using the morphological matrix because the accessory tibial spiracles—a diagnostic synapomorphy of the superfamily—cannot be observed in the 3D reconstruction of this fossil’s appendages (Garwood et al., 2011). For this reason, the inclusion of *Macrogyion cronus* in Phalangioidea was enforced as a topological constraint in the total evidence dating analysis. Similarly, the placement of *Mesobunus dunlopi* was constrained with respect to the remaining Sclerosomatidae, due to the unambiguous and observable genitalic morphology of this fossil (Giribet et al., 2012b).

**FIGURE 3:**
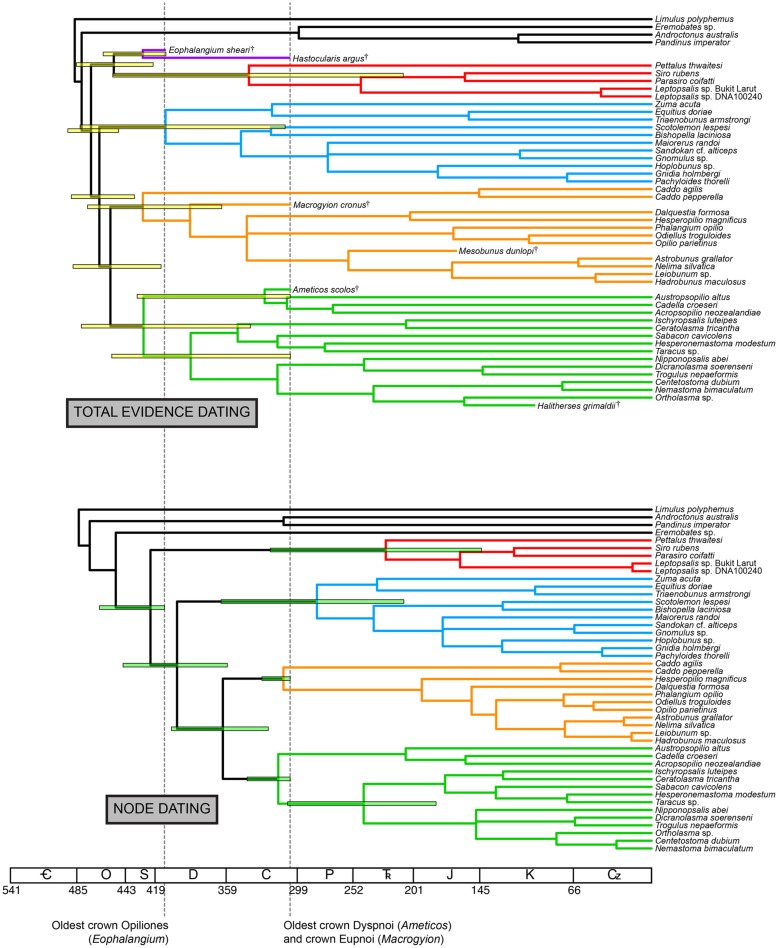
**Above: chronogram from total evidence dating under the independent gamma rates model.** Below: chronogram from node dating of the total evidence data set (without fossils) under the independent gamma rates model. Bars on nodes indicate 95% highest posterior density intervals for nodes of interest. Colors in branches correspond to suborders, as in **Figure 2**.

Total evidence dating under an independent gamma rates model recovered a chronogram with significant variance in estimated divergence times for derived nodes (**Figure 3**). Ages of major clades [with 95% highest posterior density intervals (HPD) in parentheses] were estimated as follows (**Table 2**): Opiliones: 473.2 Ma (449.9–492.7 Ma); Tetrophthalmi + Cyphophthalmi: 454.0 Ma (420.8–462.9 Ma); Phalangida: 466.2 Ma (436.4–489.8 Ma); Palpatores: 456.8 Ma (414.0–488.4 Ma); Tetrophthalmi: 429.5 Ma (410.0–462.9 Ma); Cyphophthalmi: 339.9 Ma (209.6–454.8 Ma); non-synthetonychiid Laniatores: 410.3 Ma (309.4–482.4 Ma); Eupnoi 429.5 Ma (362.8–476.2 Ma); Dyspnoi: 428.9 Ma (338.6–481.3 Ma). Large variance in the HPD of basal cyphophthalmid divergence time is the result of variable placement of Tetrophthalmi along the internode between the MRCA of Opiliones and the MRCA of Cyphophthalmi in the *a posteriori* sample of trees.

**Table 2:** Comparison of median ages and 95% highest posterior density intervals for key nodes (in millions of years).

	Total evidence dating			Combined matrix node dating			Phylogenomic matrix, LN model			Phylogenomic matrix, UGAM model		

	**Median age**	**95% HPD floor**	**95% HPD ceiling**	**Median age**	**95% HPD floor**	**95% HPD ceiling**	**Median age**	**95% HPD floor**	**95% HPD ceiling**	**Median age**	**95% HPD floor**	**95% HPD ceiling**
Opiliones	473.2	449.9	492.7	423.0	378.2	466.0	444.7	425.4	460.6	442.2	416.2	466.7
Phalangida	466.2	436.4	489.8	400.5	358.1	446.1	419.8	399.9	438.5	405.5	337.2	433.9
Palpatores	456.8	414.0	488.4	361.9	323.5	405.2	394.9	376.9	413.9	370.1	344.7	400.3
Cyphophthalmi	339.9	209.6	454.8	224.3	143.6	321.8						
non-synthetonychiid Laniatores	410.3	309.4	482.4	282.4	209.1	363.4	329.7	278.1	366.8	218.9	121.1	336.8
Eupnoi	429.5	362.8	476.2	310.7	305.3	328.9						
Dyspnoi	428.9	338.6	481.3	315.1	305.0	341.2						
non-pettalid Cyphophthalmi							330.9	278.7	375.9	144.3	44.3	328.2
Phalangioidea	341.7	265.4	430.9	193.9	127.3	263.2	313.2	305.1	333.3	315.7	305.3	342.8
non-acropsopilionid Dyspnoi	389.1	304.7	455.7	243.0	181.9	307.2	355.9	341.0	374.5	322.6	305.6	357.7

*Gray cells indicate inapplicability due to lack of taxonomic representation.*

### NODE DATING OF COMBINED (MORPHOLOGY AND MOLECULES) MATRIX

Node dating using the combined data matrix with fossils removed recovered a chronogram with consistently smaller median age estimates for all represented nodes, with respect to the total evidence dating (**Figure 3**). Ages of major clades (with 95% highest posterior density intervals [HPD] in parentheses) were estimated as follows (**Table 2**): Opiliones: 423.0 Ma (378.2–466.0 Ma); Phalangida: 400.5 Ma (358.1–446.1 Ma); Palpatores: 361.9 (323.5–405.2 Ma); Cyphophthalmi: 224.3 Ma (143.6–321.8 Ma); non-synthetonychiid Laniatores: 282.4 Ma (209.1–363.4 Ma); Eupnoi 310.7 Ma (305.3–328.9 Ma); Dyspnoi: 315.1 Ma (305.0–341.2 Ma). Asymmetrical *a posteriori* distributions of divergence times for crown Eupnoi and crown Dyspnoi indicate that the priors were informative in the analysis.

### NODE DATING OF PHYLOGENOMIC SUPERMATRIX

Node dating of the phylogenomic data set was conducted using CAT + GTR models in PhyloBayes, which incorporates infinite mixture models that better account for substitution rate heterogeneity (Lartillot and Philippe, 2004). Two models were explored for molecular dating: the lognormal [autocorrelated; LN] and the uncorrelated gamma multipliers (UGAM) models.

**FIGURE 4:**
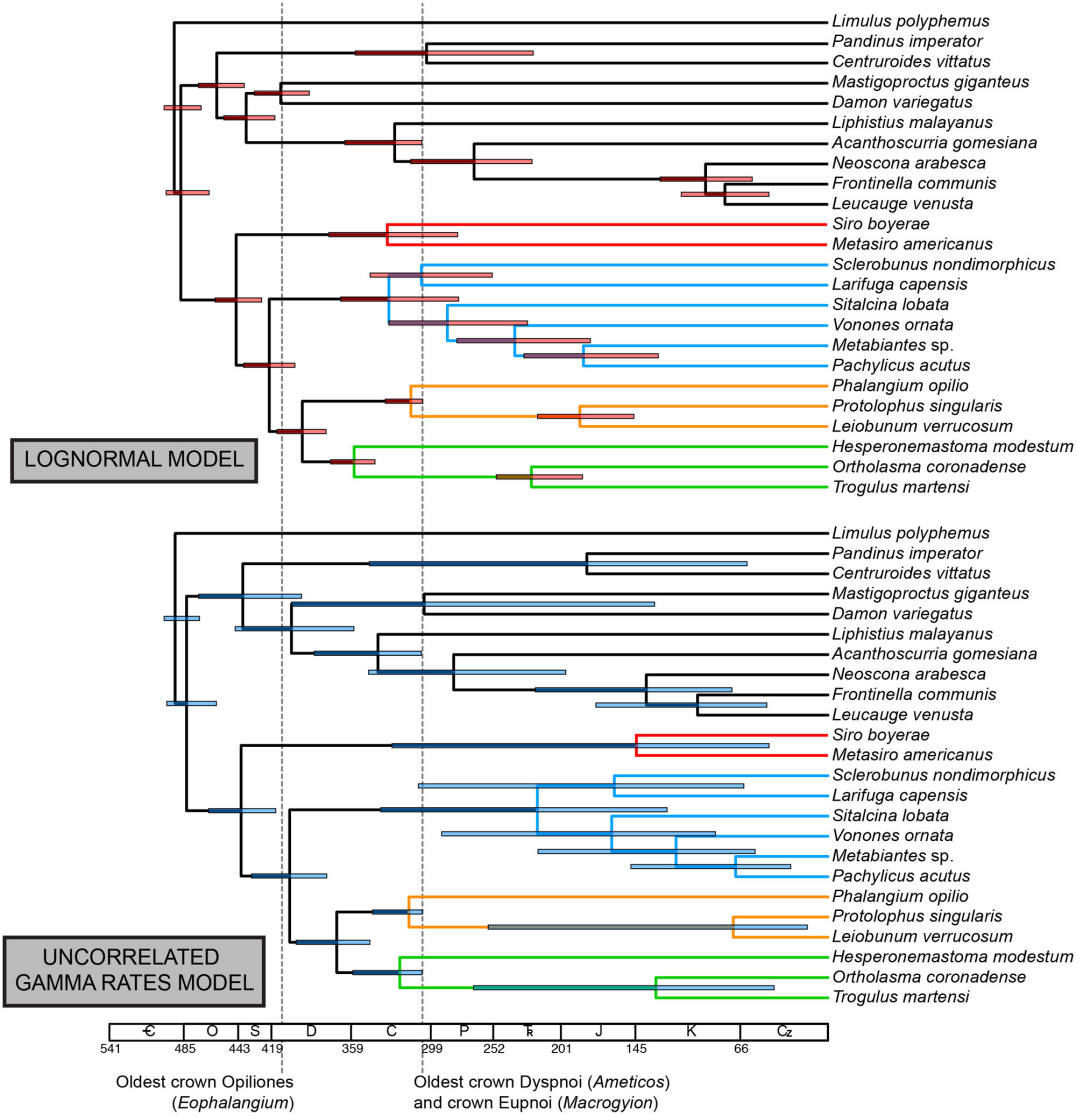
**Above: chronogram from node dating of phylogenomic matrix under the lognormal (autocorrelated) model.** Below: chronogram from node dating of phylogenomic matrix under the uncorrelated gamma multipliers model. Bars on nodes indicate 95% highest posterior density intervals. Colors in branches correspond to suborders, as in **Figure 2**.

Under the LN clock model (**Figure 4**), ages of major clades (with 95% HPD intervals in parentheses) were estimated as follows (**Table 2**): Opiliones: 444.7 Ma (425.4–460.6 Ma); Phalangida: 419.8 Ma (399.9–438.5 Ma); Palpatores: 394.9 Ma (376.9–413.9 Ma); MRCA of non-pettalid Cyphophthalmi: 330.9 Ma (278.7–375.9 Ma); non-synthetonychiid Laniatores: 329.7 Ma (278.1–366.8 Ma); Phalangioidea: 313.2 Ma (305.1–333.3 Ma); non-acropsopilionid Dyspnoi: 355.9 Ma (341.0–374.5).

The UGAM clock model (**Figure 4**) yielded the following ages of the same clades (with 95% HPD intervals in parentheses; **Table 2**): Opiliones: 442.2 Ma (416.2–466.7 Ma); Phalangida: 405.5 Ma (337.2–433.9 Ma); Palpatores: 370.1 Ma (344.7–400.3 Ma); MRCA of non-pettalid Cyphophthalmi: 144.3 Ma (44.3–328.2 Ma); non-synthetonychiid Laniatores: 218.9 Ma (121.1–336.8 Ma); Phalangioidea: 315.7 Ma (305.3–342.8 Ma); non-acropsopilionid Dyspnoi: 322.6 Ma (305.6–357.7).

## DISCUSSION

### A DATED OPILIONES TREE OF LIFE

Due to the significance of Opiliones for studies of biogeography, several authors have employed molecular dating to analyse harvestman data sets under a variety of approaches (e.g., penalized likelihood, Giribet et al., 2010; Bayesian relaxed clock methods, Sharma and Giribet, 2011; Giribet et al., 2012a). Consecutive efforts have improved upon previous counterparts, mostly through incorporation of newly discovered fossils and updated hypotheses concerning their placement (Garwood et al., 2011; Garwood et al., 2014). As an example, the oldest fossil harvestman, *E. sheari*, has previously been treated as crown-Eupnoi (e.g., Sharma and Giribet, 2011; Giribet et al., 2012a) until the first formal phylogenetic analysis including this species placed it as a stem-Cyphophthalmi with *Hastocularis argus*, the only known four-eyed harvestman (Garwood et al., 2014). Another study, also conducting node dating, treated *E. sheari* as stem-Eupnoi, but in a phylogenomic data set wherein Eupnoi were represented by two exemplars of Sclerosomatidae only; none of the remaining five families of Eupnoi were included (Hedin et al., 2012; see **Figure 1**). Therefore, Hedin et al. (2012) effectively equated the MRCA of Eupnoi with the MRCA of two Sclerosomatidae species, and the MRCA of Laniatores to the MRCA of the non-synthetonychiid laniatorids (i.e., another derived node), engendering considerable confusion in the recent literature (Table 4 of Hedin et al., 2012). The discovery of the basally branching placement of Acropsopilionidae as sister group of the remaining Dyspnoi (Groh and Giribet, 2014) also necessitates complete reevaluation of the actual MRCA of the extant dyspnoids.

The present study constitutes the first effort to analyze molecular dating in Opiliones treating all well-characterized fossil species as terminals, which has been argued to be philosophically and methodologically superior to node dating (e.g., Murienne et al., 2010; Pyron, 2011; Ronquist et al., 2012a; Wood et al., 2013). The appeal of total evidence dating stems from the consideration that the point of divergence for a given fossil along a branch can be explicitly parameterized through phylogenetic treatment of a morphological data set. While results of both approaches rely upon confident placement of fossils in a phylogeny—either *a priori* (node dating) or during the dating analysis (total evidence dating)—total evidence dating provides the singular advantage of obviating guesswork and bet-hedging with regard to fossil placement, which typically manifests as multiple analyses using alternative placements of calibration points, with often mutually exclusive alternatives (e.g., Hedin et al., 2012). Total evidence dating also enables informative use of all well-known fossils; in typical node dating analyses, only the oldest fossil corresponding to an extant clade is employed for calibration, whereas younger fossils of the constituent clade will be ignored (Ronquist et al., 2012a).

We observed that total evidence dating consistently recovered older ages than commonly used node dating methods, regardless of data set size or inclusion of morphological data (**Figures 3 and 4**; **Table 2**). This result is both intuitive and plausible; node dating approaches are limited in that they treat the age of the oldest fossil member of a clade as equivalent to the crown age of that clade. In practice, discovering the actual MRCA of a given clade in the fossil record is both highly improbable and epistemologically indefensible. Due to the incompleteness of the fossil record and well-documented artifacts associated with determining the stratigraphic range of taxa (e.g., the Signor-Lipps effect; Signor and Lipps, 1982; Marshall, 2008)—artifacts that have demonstrable consequences for molecular dating (Organ et al., 2011)—the node dating approach is an approximation and at best provides a minimum age estimate, because the actual branching point of the fossil may occur anywhere along the branch length subtending the clade.

**FIGURE 5:**
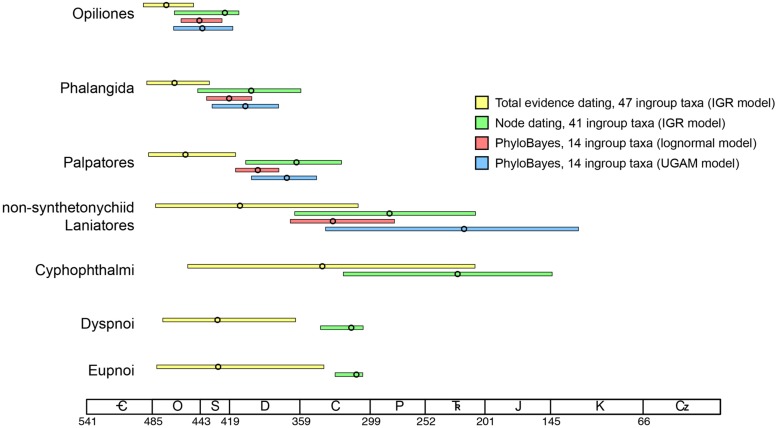
**Comparison of the four dating strategies adopted in this study, showing 95% highest posterior density intervals and median ages (circles) for nodes of interest.** Results of phylogenomic data set are restricted to the top three comparisons, as they do not capture the MRCA Cyphophthalmi (Pettalidae missing), Dyspnoi (Acropsopilionidae missing), and Eupnoi (*Caddo* missing).

Moreover, if the oldest fossil taxon of a given clade nests within that clade, and is not in fact situated along the branch subtending the clade, the inferred MRCA age of the clade will be even greater. In our data set, this phenomenon occurs throughout the Opiliones tree topology. *Eophalangium sheari*, the oldest harvestman fossil, is part of a clade sister to Cyphophthalmi (as in Garwood et al., 2014). *Ameticos scolos* and *Macrogyion cronus*, previously treated as either stem or crown group members of Dyspnoi and Eupnoi, respectively (Hedin et al., 2012), are both nested within their corresponding suborders. Finally, *Halitherses grimaldii*, treated as the oldest crown group member of the superfamily Troguloidea (Hedin et al., 2012), is in fact recovered as a derived troguloid, albeit with limited support (**Figures 1 and 2**). The consequence of these fossil placements is uniformly older ages for nodes in the total evidence dating analysis in comparison to node dating analyses (**Figures 3–5**).

Of course, the three data sets analyzed are not directly comparable due to differing taxon sets and sequence data. Yet each data set maximizes a certain type of information (fossil representation, relative influence of morphology, or extensive gene sampling). Two of our analyses also estimate the MRCA of the extant Dyspnoi (sensu Groh and Giribet, 2014) for the first time (**Figure 3**). The implementation of total evidence dating recovered an Ordovician age of Opiliones diversification (473.2 Ma), Silurian diversification of Eupnoi (429.5 Ma) and Dyspnoi *sensu* Groh and Giribet (428.9 Ma), Devonian diversification of the non-synthetonychiid Laniatores (410.3 Ma), and Carboniferous diversification of Cyphophthalmi (339.9 Ma; **Figure 3**). However, in contrast to basal divergences (e.g., Phalangida, Palpatores), estimates of subordinal divergence times are accompanied by very large HPD intervals, owing to documented topological instability of some fossil taxa (Garwood et al., 2011; **Figures 3 and 5**). The age of Cyphophthalmi is nevertheless reconcilable with previous estimates and inferred diversification in the Permian (e.g., Giribet et al., 2012a). Median ages of all three suborders of Phalangida suggest much earlier divergence times than proposed previously (e.g., Giribet et al., 2010; Sharma and Giribet, 2011; Hedin et al., 2012; Garwood et al., 2014). However, the inferred age of the non-acropsopilionid Dyspnoi—previously thought to be the MRCA of all extant Dyspnoi—is comparable to estimates from node dating (Garwood et al., 2014).

The recovery of earlier diversification dates among Phalangida’s constituent suborders is reflective of similar results in several arthropod groups. While implications of molecular dates toward inference of terrestrialization history have been discussed elsewhere (Dunlop et al., 2013; Rota-Stabelli et al., 2013; Garwood et al., 2014), we emphasize that some of the older dates obtained here were also the consequence of sampling basally branching lineages identified through recent systematic efforts, which uniquely facilitate accurate designation of MRCAs of groups of interest. Regrettably, specimens of Synthetonychiidae (the family sister to the remaining Laniatores) were not available for morphological coding, obviating their inclusion in the total evidence analysis. An ongoing effort aims to redress this shortcoming (R. Fernández and G. Giribet, personal communication).

### PHYLOGENOMICS, MODEL CONTINGENCY, AND THE POWER OF FOSSILS

Major lineages within suborders are not available among the sequenced transcriptomes of Opiliones. In the phylogenomic supermatrix analyzed, the sister group of the remaining Cyphophthalmi (Pettalidae), Laniatores (Synthetonychiidae), Eupnoi (Caddidae *sensu stricto*), and Dyspnoi (Acropsopilionidae) were all conspicuously absent. Nevertheless, we analyzed the supermatrix under two commonly deployed clock models, the lognormal (Thorne et al., 1998) and uncorrelated gamma multipliers (Drummond et al., 2006) models. The two models are grounded in different assumptions of heritability of substitution rates (Lepage et al., 2007), but in many cases have been shown to converge upon similar estimates when sufficient data (both molecular sequence data and fossil calibrators) are available. Apropos, we observed congruence in age estimates for basal nodes in both analyses that were well constrained with respect to fossil calibrations or adjacent (constituent) nodes (e.g., Opiliones, Palpatores, Phalangida; **Figure 4**). Such age estimates were also congruent with counterparts from total evidence dating (**Figure 5**), in spite of the markedly different composition of the two data sets.

By contrast, subordinal age estimates were far more variable. Phalangioidea (represented by *Phalangium opilio*, *Protolophus singularis*, and *Leiobunum verrucosum*) and the non-acropsopilionid Dyspnoi (represented by *Hesperonemastoma modestum*, *Ortholasma coronadense*, and *Trogulus martensi*) had comparable ages of basal diversification, a consequence of lower bound estimates applied to these suborders, on the basis of phylogenetic placements of *Ameticos scolos* and *Macrogyion cronus* (**Figure 2**). But median ages of the non-pettalid Cyphophthalmi (*Siro boyerae* + *Metasiro americanus*) and the non-synthetonychiid Laniatores varied radically between the two PhyloBayes analyses (**Figure 4**). The differences were not statistically significant because HPD intervals in the UGAM analysis were very large.

These results indicate that model implementation may be one major explanatory variable toward accounting for the large range of dates obtained for unbounded nodes in Opiliones. Hedin et al. (2012), using a subset of eight taxa analyzed here, obtained a range of dates by implementing various combinations of fossil calibrations, many of them loosely applied with respect to actual MRCAs (as discussed above). In that study, a single Cyphophthalmi was available, and so no internal dating was possible within mite harvestmen. But the dating of Laniatores [to which no fossil calibrations were applied either by Hedin et al. (2012) or in this study] exhibited the broadest range of estimates, from one analysis to the next. Our results indicate that this variance can stem from a combination of different model assumptions and the absence of bounds on nearby nodes (fossil calibrations).

Tackling the sensitivity of shallow nodes to model choice is largely a matter of taxonomic sampling, both fossil and extant. While we endeavored to include as many well-known harvestmen fossils as possible, we were limited by inaccessibility of several key fossils for study (e.g., the Cretaceous cyphophthalmid *Palaeosiro burmanicum*), and thus did not code these in the morphological matrix. Similarly, some highly derived Cenozoic fossils were not coded in the matrix either; the diversity of Laniatores in particular necessitates sampling potentially hundreds or even thousands of extant species in a total evidence analysis in order to represent those nodes (genera) near which Miocene amber specimens are anticipated to diverge (Sharma and Giribet, 2011, 2012).

The greatest congruence in divergence time estimation across analyses and data sets occurs for nodes that are well constrained by fossil exemplars, an intuitive result. This study thus reinforces the significance of fossil discovery and systematic paleontology toward reducing uncertainty in molecular dating, and specifically over the impetus to improve precision by adding sequence data. While augmenting sequence data is anticipated to improve guidance of model selection, the greatest gains to precision in molecular dating remain attributable to discovery and algorithmic treatment of fossil taxa (Pyron, 2011; Ronquist et al., 2012a). While consensus accrues with regard to basal Opiliones divergence dates, the absence of pre-Cenozoic Laniatores and pre-Mesozoic Cyphophthalmi fossils specifically engenders great variance in estimated ages of these suborders’ basal diversification (Sharma and Giribet, 2011; Hedin et al., 2012; Garwood et al., 2014). As a consequence, these node ages remain imprecise (**Figure 3**) and highly sensitive to model choice, regardless of amount of sequence data (**Figure 4**).

## PERSPECTIVES

Dating the tree of life has become a common enterprise for better understanding key evolutionary processes, such as terrestrialisation of arthropods (e.g., Rota-Stabelli et al., 2013), or to interpret past rates of evolution (Lee et al., 2013), among many others. Due to its recent implementation in model-based approaches (Murienne et al., 2010; Pyron, 2011), relatively little effort has been dedicated to date toward evaluating total evidence dating with respect to other methodologies. Here we demonstrated that total evidence dating consistently yielded older and more plausible divergence time estimates than node dating in the explored data set.

Uncertain fossil placements and the lack of derived fossils for key groups beleaguer precise divergence time estimates for shallow nodes (e.g., suborders; **Figures 4 and 5**). Improving precision of molecular age estimates for shallow nodes will require sampling a large number of relatively young calibration points, as well as numerous derived lineages within the suborders. We proffer two critical imperatives for future dating practices of diverse, understudied groups: (a) robust phylogenetic treatment of newly discovered fossils (e.g., Garwood et al., 2014), and (b) rigorous reassessment of systematics of extant lineages, many of which may prove integral to estimation of MRCAs of higher clades (e.g., Groh and Giribet, 2014).

## Conflict of Interest Statement

The authors declare that the research was conducted in the absence of any commercial or financial relationships that could be construed as a potential conflict of interest.
